# The Effect of Disinfectants Absorption and Medical Decontamination on the Mechanical Performance of 3D-Printed ABS Parts

**DOI:** 10.3390/polym13234249

**Published:** 2021-12-03

**Authors:** Diana Popescu, Florin Baciu, Catalin Gheorghe Amza, Cosmin Mihai Cotrut, Rodica Marinescu

**Affiliations:** 1 Faculty of Industrial Engineering and Robotics, University Politehnica of Bucharest, 060042 Bucharest, Romania; diana.popescu@upb.ro (D.P.); acata1@camis.pub.ro (C.G.A.); 2 Faculty of Materials Science and Engineering, University Politehnica of Bucharest, 060042 Bucharest, Romania; cosmin.cotrut@upb.ro; 3 Department of Orthopedics, University of Medicine and Pharmacology Carol Davila, 020021 Bucharest, Romania; rodicamarinescu@ymail.com

**Keywords:** 3D printing, medical decontamination, disinfection, medical device, ABS, absorption, mechanical performance

## Abstract

Producing parts by 3D printing based on the material extrusion process determines the formation of air gaps within layers even at full infill density, while external pores can appear between adjacent layers making prints permeable. For the 3D-printed medical devices, this open porosity leads to the infiltration of disinfectant solutions and body fluids, which might pose safety issues. In this context, this research purpose is threefold. It investigates which 3D printing parameter settings are able to block or reduce permeation, and it experimentally analyzes if the disinfectants and the medical decontamination procedure degrade the mechanical properties of 3D-printed parts. Then, it studies acetone surface treatment as a solution to avoid disinfectants infiltration. The absorption tests results indicate the necessity of applying post-processing operations for the reusable 3D-printed medical devices as no manufacturing settings can ensure enough protection against fluid intake. However, some parameter settings were proven to enhance the sealing, in this sense the layer thickness being the most important factor. The experimental outcomes also show a decrease in the mechanical performance of 3D-printed ABS (acrylonitrile butadiene styrene) instruments treated by acetone cold vapors and then medical decontaminated (disinfected, cleaned, and sterilized by hydrogen peroxide gas plasma sterilization) in comparison to the control prints. These results should be acknowledged when designing and 3D printing medical instruments.

## 1. Introduction

The medical sector benefits from the Additive Manufacturing (AM) technology’s ability to provide customization (surgical guides and instruments [1,2,3] and anatomical replicas [4]) and to sustain delocalized and digitized manufacturing—very important aspects in situations such as SARS-COV-19 pandemic lockdown [5,6] or in remote areas, war zones, and outer space [7,8]. Moreover, AM is characterized by a short supply chain [9], being capable of rapidly responding to the need of critical medical spare parts [10] or personal protective equipment (PPE) [6,10].

AM technology transforms a digital object into a physical one by superposing layers of material. It does not require specific tools, molds, or fixtures, while the affordability and the availability of equipment (in the case of AM based on the material extrusion process, also known as 3D printing—3DP) have made it a ‘democratic’ manufacturing technology. However, this popularity should not be misleading. To 3D print functional parts able to reliably and efficiently operate in real conditions, extensive knowledge and testing is required for optimizing prints’ design and parameter settings for specific uses and environments. As such, in the medical field, there is a need for understanding the relationship between the printing parameters and the behavior of 3D prints subjected to specific medical decontamination procedures, and thus to validate their viability as reusable medical instruments, PPE, or other devices.

AM applications in medicine have been reported since the beginning of the 1990s. 3D prints are currently used in pre-planning, rehearsing, and performing different medical tasks, for communication, or training purposes. When used in direct contact with the patient, 3D-printed objects should be decontaminated (meaning disinfection, cleaning/washing, and sterilization) using the typical protocols in healthcare facilities [11]. For polymer-based 3D prints, the literature presents several studies on the effect of different sterilization methods (autoclave, ethyleneoxide gas, hydrogen peroxide gas plasma, and gamma radiation) over the sterility, mechanical properties, and dimensional and form accuracy [12,13,14,15,16,17]. For instance, Popescu et al. assessed the influence of natural aging (shelf life) and multiple hydrogen peroxide gas plasma sterilization on the stiffness and strength in tension and flexion, reporting no significant changes in the mechanical behavior of sterilized 3D-printed ABS samples [12]. Rankouhi et al. analyzed the impact of gamma irradiation on ABS (irradiated filament vs. irradiated 3D-printed ABS) on the mechanical properties showing the suitability of this method for surgical instruments sterilization [14]. Rankin et al. investigated the use of glutaraldehyde for sterilization, proving that it does not significantly influence the tensile strength of a 3D-printed PLA (polylactic acid) army retractor [17].

However, there are only few reported studies focused on the effects of cleaning and disinfection over the 3D prints performances. Török et al. found no influence of the disinfection solution (4% Gigasept^®^, immersion for 60 min) and autoclave on the dimensional accuracy, hardness, and flexural and compressive strength of dental surgical guides (PolyJet 3D Printing technology and medical grade resin) [18]. Fleischer et al. investigated the impact of disinfection and sterilization on PLA samples’ mechanical behavior, mass, and surface quality in relation with different printing settings [19]. Samples were soaked in chlorine and Cidex OPA solutions following two decontamination procedures. The treatments changed PLA prints behavior from ductile to brittle, and increased the samples mass, proving the absorption of the chemical solutions. The authors noted the necessity of minimizing the surface porosity that favors the accumulation of disinfectant within the 3D-printed medical devices and increases the risk of contaminating the patients. The same observation on safety is made by Singh et al. referring to the possibility of contaminated droplets to enter and remain within the 3D print [20]. Even for 3D prints with full infill density, internal voids (air gaps or pockets) are formed between the filament threads and between the shell and the infill threads (raster to perimeter voids) [21], while the layers addition might create defects in the form of external pores communicating with the interior of the part, thus enhancing fluid intake. In this sense, Moreno et al. reported that the fluid absorption rates are larger for 3D-printed specimens from PLA and PETG than for the corresponding raw materials [22]. 3D prints permeation to air was also studied, Gordeev et al. optimizing the parameter settings to improve sealing [23]. Vaňková et al., in the context of manufacturing PPE for SARS-COV-19 pandemic, studied the effect of ethanol, isopropanol, and sodium hypochlorite on the 3D-printed PLA face masks [24]. PPE made of PLA were rinsed in pure acetone for smoothing the surface and closing the external pores as shown in SEM (scanning electron microscopy) investigations. Vapor acetone is often reported as surface treatment for improving the prints surface quality [25,26] and for sterilization in medical applications [27]. However, no study was found on the combined effect of the acetone treatment and medical decontamination.

As the intrinsic porosity of 3D prints corroborated the infiltration of disinfection solutions or patient body fluids within the reusable 3D-printed instruments might pose safety issues, it is necessary to evaluate the correlation between the fluid absorption rate and the printing parameters settings, as well as the influence of the absorbed fluids and medical decontamination on the prints’ mechanical performance. Therefore, the literature examined for this study also included studies on 3D prints water absorption as function of layer thickness, raster angle, or build orientation [28,29,30,31,32], and on the tensile strength evaluation of water submerged and hydrated samples [33]. It could be noted that the majority of the reported research is focused on PLA specimens. Additionally, all studies agree that the porosity of 3D prints enhances fluid absorption. Therefore, this permeability should be acknowledged and controlled by using appropriate parameter settings and post-processing treatment, for a safe reuse of the 3D-printed medical devices. Moreover, the existing research considers static water absorption to be the case of the samples being immersed in fluid until saturation. However, the medical practice also includes dynamic procedures such as washing, scrubbing, and rinsing after soaking in disinfectant, which take place for varying duration depending on the type of cleaning substances.

In this context, the following investigations were identified as important for the field and are pursued in this study:

- Disinfectant absorption rate dependency of layer thickness, number of perimeters and infill pattern for 3D-printed ABS samples immersed in disinfectant solution. This will answer the research question 1: Is there a set of 3D printing parameters that can improve the sealing and reduce the fluid infiltration?
- Compressive and tensile strengths of 3D-printed ABS disinfected specimens. This will answer the research question 2: Are the mechanical performance of 3D-printed specimens degraded after immersion in disinfectants and sterilization?
- Cold vapor acetone-based surface treatment influence on the fluid absorption rate and on the mechanical behavior of a 3D-printed surgical retractor. This will answer the research question 3: Is the acetone treatment a solution for sealing the 3D-printed ABS reusable medical instruments and not degrading the mechanical performances in corroboration with the medical decontamination?

## 2. Materials and Methods

Three experiments were conducted for answering the above research questions. In brief, these experiments are (name/type of samples/investigations):

- Experiment 1/cubes/absorption and compression test;
- Experiment 2/tensile samples/absorption and tensile test, SEM investigations;
- Experiment 3/decontaminated medical retractors/absorption test, tension-bending combined loading test.

### 2.1. 3DP Specimens and Surgical Retractors

All prints were manufactured from white ABS filament (Gembird Ltd., Almere, The Netherlands) on Creality CR10 3D printer (Shenzhen Creality 3D technology Co., Ltd. Shenzhen, China). Cura 4.6 (Ultimaker, Inc., Utrecht, The Netherlands) was used as slicer. For each experiment, three samples were manufactured and tested to ensure repeatability. 

In Experiment 1, cubes of 12 mm length were 3D printed (Table 1) for assessing the disinfectant absorption rate dependency on printing parameter settings (Table 2) in a full factorial design of experiments (research question 1). The saturated specimens were also tested for compression (research question 2). The values of the fixed parameters (temperatures, printing speed, flow rate, etc.) were selected based on authors’ previous experience with the 3D printer and material for producing samples with no visible defects in terms of layers adhesion or warpage. An infill density of 70% was set for all prints based on the available data for 3D-printed medical instruments [17,34]. The variable parameters were number of perimeters, infill pattern, and layer thickness. In order to investigate if the fluid infiltration depends on the infill patterns, two geometries were considered: ±45° lines and gyroid. Lines pattern is one of the most common and studied infill pattern used in 3DP, while gyroid provides a nearly isotropic behavior [19,35], useful for manufacturing functional parts subjected to loads from different directions. Leite et al. conducted research on the water absorption dependence on build orientation and raster angle for ABS P400 specimens (0.254 mm layer thickness), also analyzing samples’ compressive and tensile properties [29]. With respect to the raster angle, their experiments confirmed Vincente et al. results [28], i.e., 0°/90° raster angle leads to more porosity and more absorbed water. This is the reason for selecting ±45° as raster angle for all the 3D prints in this research.

**Figure 1:**
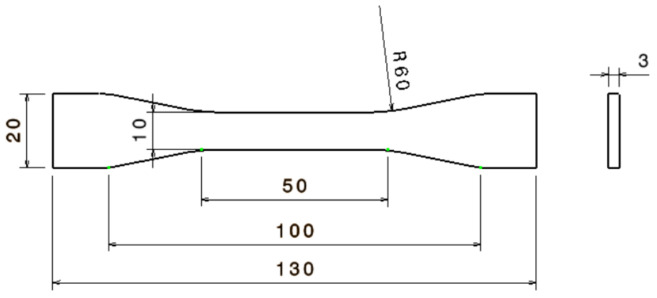
Type IV tensile specimen dimensions.

In Experiment 2, tensile specimens (according to ASTM D638) were 3D printed (Figure 1). The results of the absorption tests from Experiment 1 allowed identifying the parameters settings that provide the best and the least sealing ability. These settings were used for manufacturing the tensile specimens (Table 3). Keeping these specimens in disinfectant until saturation was considered relevant for investigating the potential degradation of mechanical properties caused by the disinfectant action on ABS prints (research question 2). Twelve specimens were manufactured in total, six for each set of conditions and control.

**Figure 2:**
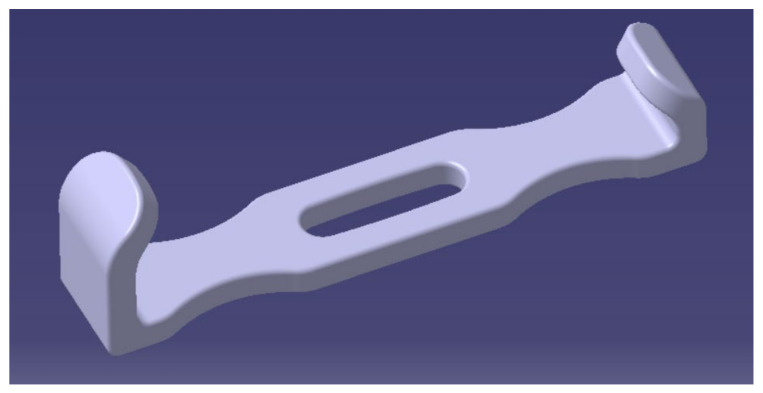
Parker Langenbeck surgical retractor.

In Experiment 3, mechanical tests (tension-bending combined loading) were performed on six 3D-printed ABS surgical retractors (Figure 2) produced using the parameters settings which offer the least sealing ability after 30 min of soaking in disinfectant and the shortest manufacturing time. The purpose of this investigation was to check if, in the fast manufacturing condition, the retractors are suitable for use after post-treatment and decontamination (research question 3). Surface treated retractors were also weighted before sterilization to assess the efficiency of the acetone post-processing. Three of these medical devices were surface treated with acetone cold vapors and then subjected to medical decontamination with disinfectant solutions and hydrogen peroxide sterilization. Three retractors were not post-processed or decontaminated, being used as control group.

### 2.2. Medical Decontamination

Medical decontamination (disinfection, cleaning/washing, and sterilization) is a complex process, critical in achieving instruments free of any kind of soil-blood or tissue/bone debris. It depends on several factors such as the chemical activity of the cleaning solution, the type of cleaning maneuver (manual or mechanical) and the geometry of the item to be decontaminated.

There are various products used for cleaning surgical instruments, requiring different durations for their action. In this research, two products were used: Aniosyme XL3 (Ecolab, Saint Paul, MN, USA) and Sekusept (Laboratories Lezennes, Lezennes, France, Fr). Aniosyme is a high-level detergent with enzymatic activity (composition: ammonium quaternary carbonate, non-ionic surfactant, and enzyme complex, 0.5% dilution). Sekusept is used for cleaning and high disinfection surgical instruments and endoscopes (composition: sodium perborate and tetraacetylenediamine, 1% dilution).

The specimens in Experiments 1 and 2 were soaked in disinfectants until saturation (i.e., until no significant mass change caused by the fluid infiltration occurred between two consecutive measurements). For the 3D-printed retractors in Experiment 3, a typical decontamination process was followed in a local hospital. The retractors were soaked in Aniosyme for 30 min and their surfaces were scrubbed using a soft-nylon brush, followed by rising in water. Then, they were immersed in Sekusept for 15 min. Rinsing was also necessary at the end of this process. Then, the 3D-printed instruments were dried and sterilized. Hydrogen peroxide gas plasma sterilization was performed on retractors using Sterrad equipment with a 45 min cycle program under 134 °C and 0.223 MPa (2.2 atm) [12].

### 2.3. Post-Processing Using Acetone Cold Vapors

As mentioned, the surgical retractors were manufactured using the printing parameters providing the least sealing and fastest manufacturing time, and then exposed to cold acetone vapors for 45 min [26]. This method of treatment was shown in the literature to determine a more gradual and uniform smoothing than hot acetone vapor or immersion, and with less dimensional degradation. Moreover, it is easier and safer to perform [36,37].

### 2.4. Experimental Tests

#### 2.4.1. Experiment 1: Disinfectant Absorption Rate and Effect on Samples Compression Strength

For the absorption tests, the cubes were repeatedly immersed in disinfectant at room temperature (22–24 °C) and their mass was measured. The first measurement was performed on dry specimens and the second measurement was made 10 min after by removing the cubes from the solution and placing them on a dry cloth for eliminating the excess of disinfectant. In the first two and a half hours, the cubes were weighed every half an hour. Then, the measurements were performed after 5 h, 7 h, 10 h, 24 h, 30 h, 48 h, 72 h, and 7 days. In total, the samples were weighed fifteen times (minutes: 0, 10, 30, 60, 90, 120, 150, 300, 420, 600, 1440, 1800, 2880, 4320, and 10,080).

The compression testing was performed on saturated specimens using an Instron 8872 Universal Test Machine (Instron Inc., Noorwood, MA, USA) with a constant compression velocity of 1 mm/min until the specimens’ fracture.

#### 2.4.2. Experiment 2: Disinfected Samples Tensile Property Assessment

The tensile specimens were immersed in disinfectant until saturation. Methylene blue was added in the solution for evaluating the fluid penetration depth by observing the coloring of the fracture surfaces. Specimens were tested using an Instron 8872 Universal Test Machine (Instron Inc., Kawasaki, Japan) equipped with a load cell of 25 kN. The testing speed was set at a rate of 1 mm/min and the extensometer gauge length was 25 mm.

Phenom ProX scanning electron microscope (SEM) (Phenom World, Eindhoven, The Netherlands) was used for investigating if the specimens fracture surfaces show any specific features which might be caused by the disinfectant action.

#### 2.4.3. Experiment 3: Post-Processing Influence on the Fluid Absorption and on the Mechanical Performance of a Surgical Retractor

**Figure 3:**
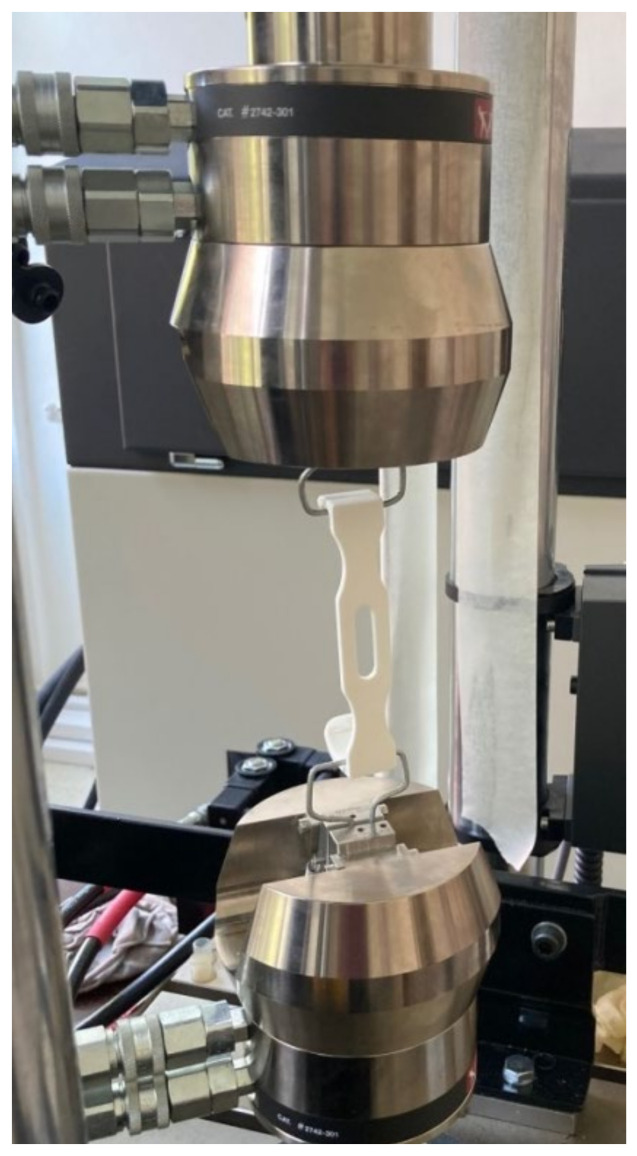
Experimental set-up for the testing of surgical retractors.

Three surgical retractors post-treated with cold acetone vapors were subjected to a typical decontamination cycle in a clinical hospital, as described in Section 2.2. Treated retractors’ mass was measured, after soaking and cleaning in disinfectants, before sterilization. The retractors’ mechanical behavior was evaluated using the same equipment as in Experiment 2 (Figure 3), in an experimental set-up similar with the one used by Chen et al. [38].

### 2.5. Microscopic Examination

After Experiment 2, randomly selected tensile samples were cut with a sharp chirurgical blade to provide small representative pieces for microscopic investigation, and then the top surfaces and the fracture surfaces of decontaminated specimens were SEM examined referring to the control specimens.

## 3. Results and Discussions

### 3.1. Experiment 1: Disinfectant Absorption Rates and Samples Compression Strength

**Figure 4:**
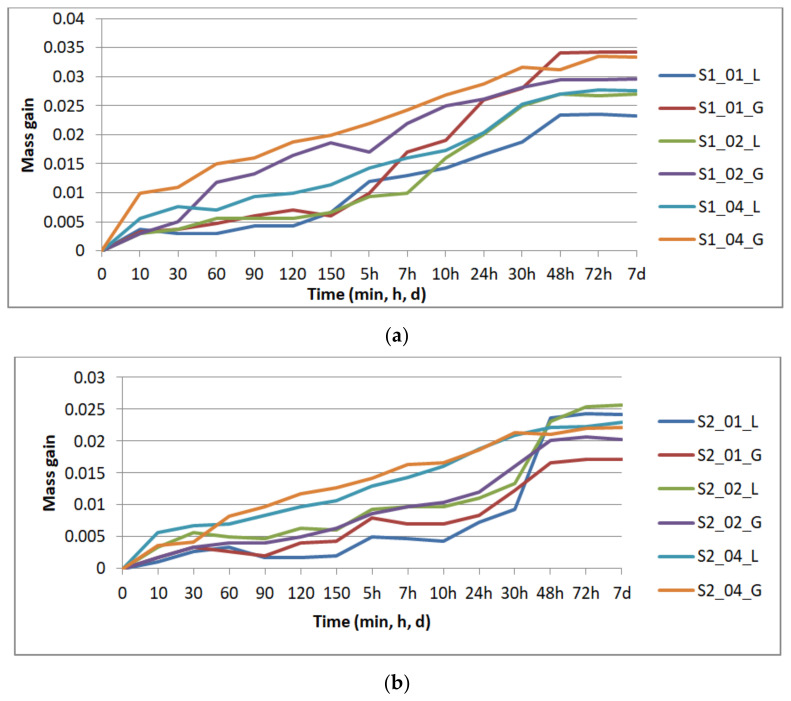

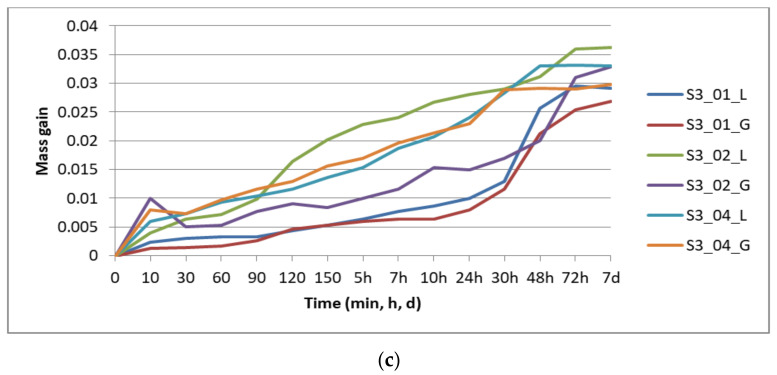
Disinfectant absorption for specimens 3D-printed with one perimeter (**a**), two perimeters (**b**), and three perimeters (**c**).

Figure 4 presents the results of the specimens’ mass gain by repeated immersion in disinfectant, graphically grouped according to the number of perimeters: one perimeter (Figure 4a) two perimeters (Figure 4b), and three perimeters (Figure 4c), for all the layer thicknesses (0.1 mm, 0.2 mm, and 0.4 mm) and the two analyzed infill patterns (lines—L, gyroid—G). 

The mass gain was evaluated as the difference between the sample mass at time t (m_t_) and its dried mass (m_dry_): m_t_ − m_dry_.

The absorption rate (CI) was also calculated: CI (%) = 100 × (m_t_ − m_dry_)/m_dry_.

Saturation occurred after 48 h for the specimens with one and two perimeters, and after 72 h for the specimens with three perimeters. The compression testing was performed on the specimens removed from disinfectant after seven days.

**Figure 5:**
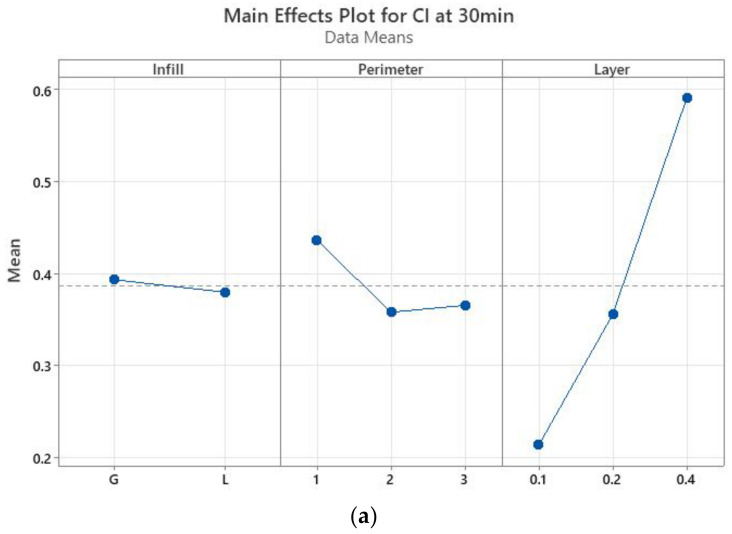

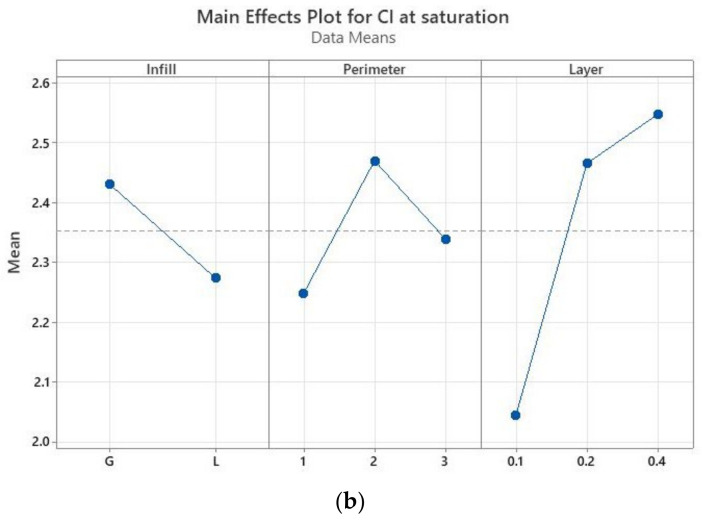
Main effect plots for the absorption rate: (**a**) after 30 min of immersion, (**b**) at saturation.

The main effects plots (Minitab, Minitab UK) are represented in Figure 5 for CI. These graphs show that CI at 30 min is not influenced by the infill pattern (Figure 5a), but by the layer thickness, while there is no significant difference between the specimens with two or three perimeters. At saturation (Figure 5b), the layer thickness is again the most influential parameter—the higher the thickness, the higher the absorption rate. The infill pattern has the least influence, while one perimeter ensures a smaller absorption rate in comparison to two and three perimeters. If the samples with one perimeter are providing the least sealing after 30 min of immersion, at saturation they ensure the best sealing. This means that disinfectant infiltrates the quickest in the prints with one perimeter, the specimens reaching saturation faster than in the other conditions as confirmed by the results displayed in Figure 4.

Thus, the samples with 0.1 mm layer thickness ensured the best sealing, followed by the specimens with 0.2 mm and then 0.4 mm layer thicknesses. This confirms the findings of Ayrilmis et al. who analyzed the influence of layer thickness (0.05 mm, 0.1 mm, 0.2 mm, and 0.3 mm) on the water absorption of 3D-printed wood/PLA specimens at 0° raster orientation [30]. However, the result is not in agreement with Vincente et al. who concluded that 0.2 mm layer thickness determines the least water absorption of ABS samples in comparison to 0.1 mm layer thickness [28].

**Figure 6:**
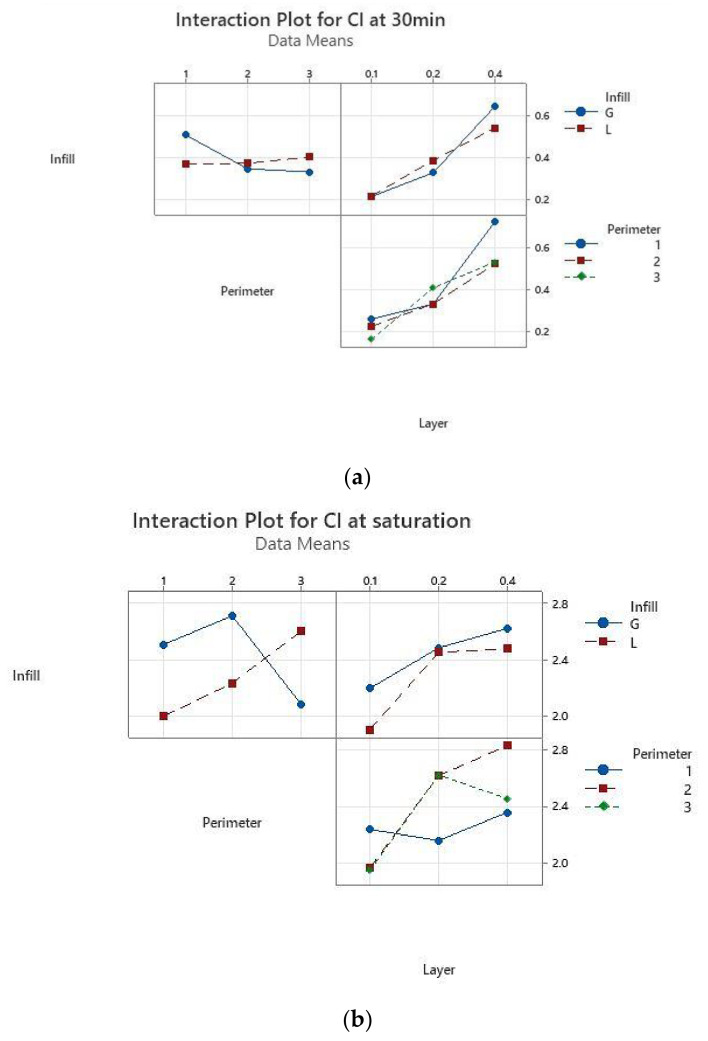
Interaction plots for the absorption rate: (**a**) after 30 min of immersion, (**b**) at saturation.

The interaction for layer thickness by perimeter is significant both after 30 min of immersion and at saturation, while the interaction for layer thickness and infill pattern has a moderate significance, as shown in Figure 6.

The results of the absorption tests after 30 min of immersion in disinfectant showed that the samples with 0.1 mm layer thickness and two perimeters absorb less disinfectant. At the other end are the samples with 0.4 mm thickness and one perimeter, which absorb the largest quantity of disinfectant. This information was used for manufacturing the required tensile specimens for Experiment 2 (Table 3), as the medical instruments are usually soaked in disinfectant for 20–30 min (depending on the type of disinfectant solution).

The results of the compression tests are presented in Table 4. By comparing the results in pairs (e.g., S1_01_L to S1_01_G; S2_02_L to S2_02_G), it can be observed that the saturated specimens with linear infill have a higher modulus of elasticity than the specimens with gyroid infill, regardless of the number of perimeters and layer thickness. Additionally, the smaller the layer thickness, the higher are the modulus of elasticity and the compressive yield strength, regardless of the number of perimeters and infill pattern, as noted in a recent study conducted on dry specimens, 3D printed at 100% infill density [39]. Our research findings suggest that when selecting the 3DP parameters for a medical instrument or device subjected to disinfection (and which can absorb disinfectant agents), the linear infill provides more stiffness than the gyroid infill (if the compression load is applied along the print build direction). 

There is research investigating the compression behavior for 3D-printed samples subjected to water immersion [29]. These were treated with polyurethane, acrylic coating, and acetone (45%, 50%, 70%, and 80%) with the purpose of analyzing the effect of the post-processing treatments on the compressive strength, CI, and open porosity. The post-processing treatment by immersion in acetone is decreasing the compressive properties, while the acrylic coating proved to be efficient in porosity reduction while at the same time maintaining 3D prints mechanical properties. Additionally, the results showed that only the 80% acetone solution and 3h treatment reduced both the open porosity and CI. However, Leite et al. also note that the acetone treatment by immersion is not effective due to samples geometry modifications [29]. As it was clear that longer exposure to acetone affects samples dimensional stability, cold vapor acetone was used instead of immersion in the current research.

### 3.2. Experiment 2: Disinfected Samples Tensile Property Assessment

**Figure 7:**
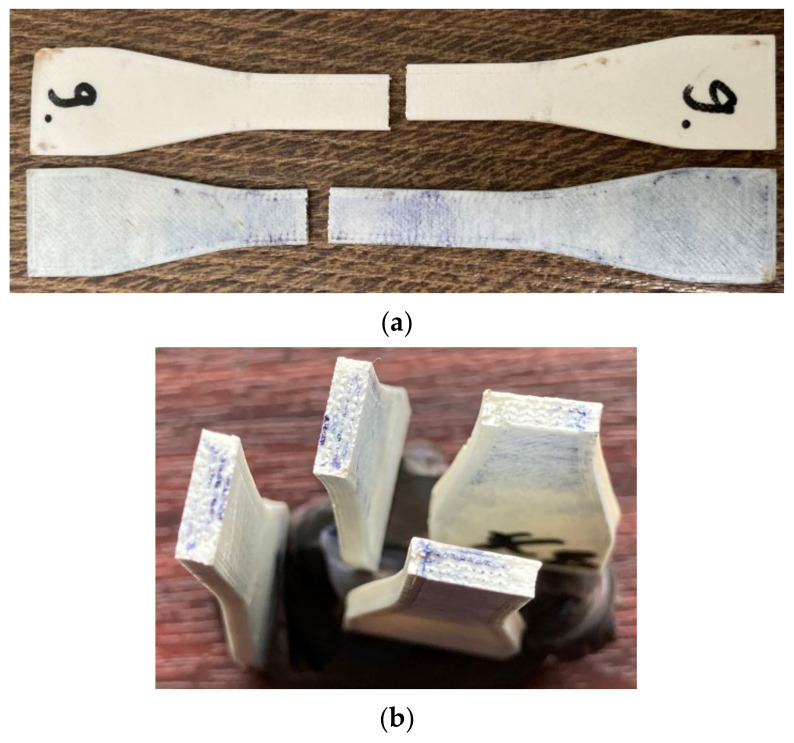
Disinfectant infiltration within tensile specimens: (**a**) infiltrated specimen vs. control specimen; (**b**) disinfectant infiltrations in 3D-printed specimens.

Figure 7 presents images proving the disinfectant infiltration within different specimens (methylene blue coloring). The disinfectant infiltrated up to the infill area, where more pores were present due to the 70% infill density. No similar research was found for analyzing this observation in connection with other 3D printing materials or process parameters settings.

**Figure 8:**
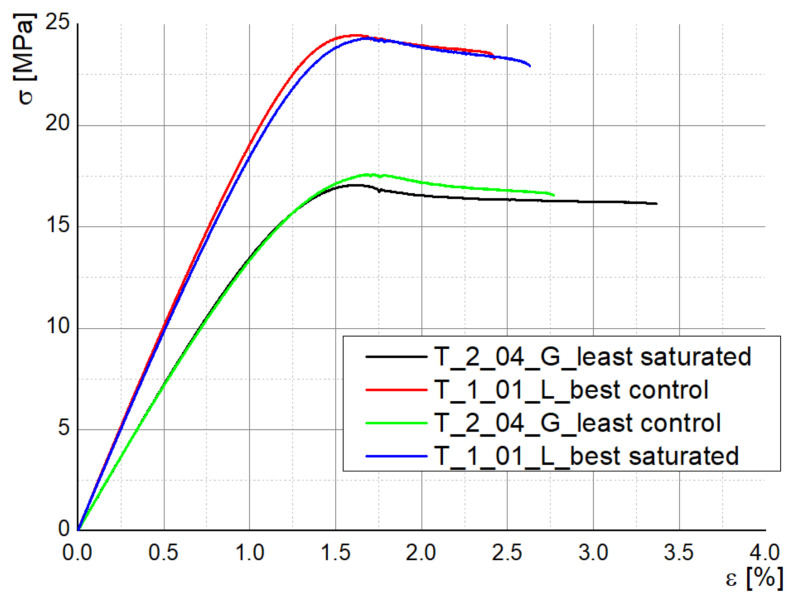
Stress–strain curves for saturated and control tensile specimens.

The stress–strain curves for the specimens tested in Experiment 2 are almost coincident when comparing the disinfected and control samples for each parameters combination (best sealing and least sealing), which show that the employed disinfectants do not degrade the bulk properties of the material (Figure 8). Young modulus shows a slight decrease only for the samples manufactured with the best sealing properties: 2013.94 MPa (T_1_01_L control) vs. 1961.88 MPa (T_1_01_L disinfected), 1439.89 MPa (T_2_04_G disinfected) vs. 1432.99 MPa (T_2_04_G control). In the same sense, Kim et al. investigated the maximum absorption rate for 3D-printed ABS specimens and its effect on the tensile strength and reported that both moisture and temperature are reducing the values of tensile strength and Young’s modulus, the water effect being more significant [32].

**Figure 9:**
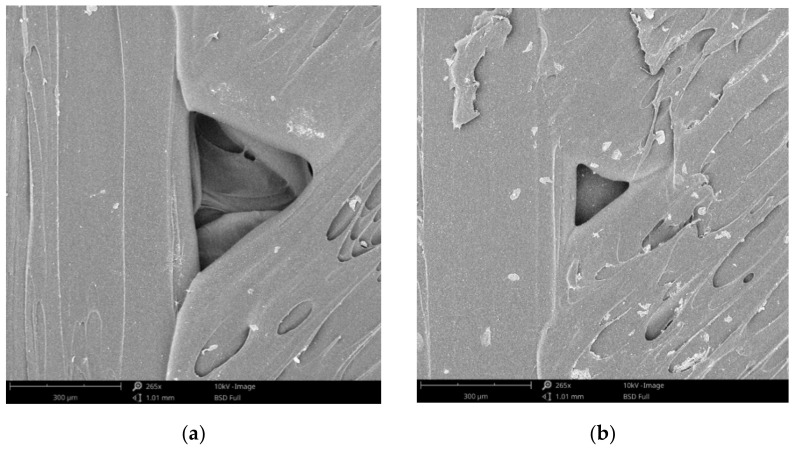

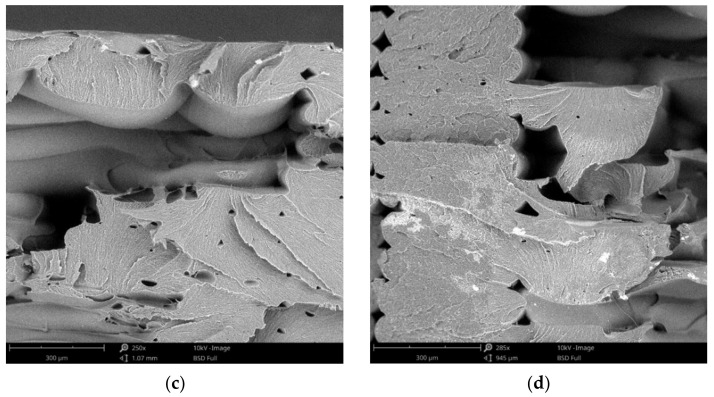
SEM images of disinfected (**a**,**c**) and control specimens (**b**,**d**) (**a**,**b**—top view; **c**,**d**—fracture surface).

Representative SEM images (top view and fracture surface) of disinfected and control specimens and are presented in Figure 9, and the following can be observed:

- Perimeter to raster void and porosity on the external surface of control specimen (Figure 9a);
- Crystals formed on the external surfaces after drying the disinfection medium (Figure 9b);
- Fracture surface of control specimen (Figure 9c);
- Fracture surface of disinfected specimens (Figure 9d).

The absorption of the disinfectant is directly related to the porosity of the 3D prints. SEM investigations showed the formation of raster to perimeter voids (as in Figure 9a), thorough research in this sense being conducted by Wang et al. [40], as well as by Popescu et al. [12] by means of industrial computer tomography.

No degradation of the material was noticed as result of disinfectants action after SEM examination. Therefore, no supplementary investigation on material degradation was conducted.

### 3.3. Experiment 2: Influence of Acetone Treatment on the Mechanical Properties of a Surgical Retractor

**Figure 10:**
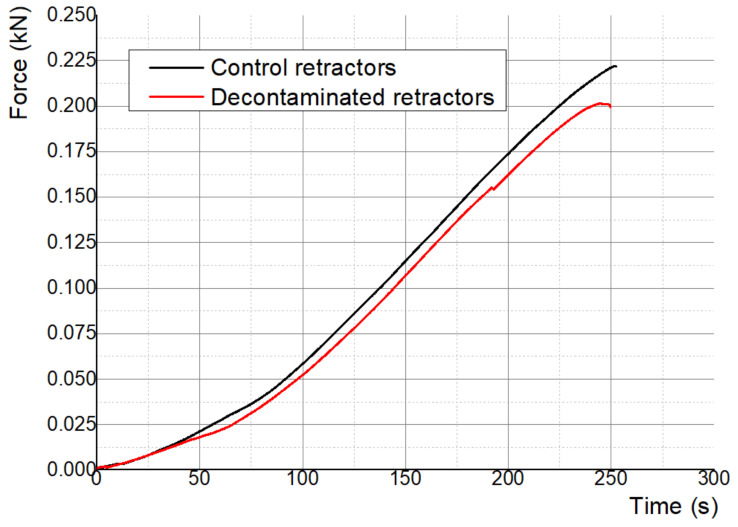
Mechanical test results for retractors.

Figure 10 presents the testing results for the surgical retractors. The mechanical properties of the acetone treated retractors subjected to medical decontamination are inferior to the mechanical properties of the retractors used as control. Cold acetone vapor treatment effect on the ABS samples mechanical properties (after 1 h and 2 h of exposure) was investigated by Gao et al. [41]. Three build orientations were analyzed, the acetone post-processing decreasing the strength and increasing elongation at failure for x–y orientated specimens. Our study results confirm these findings. It should be noted that the acetone processed retractors, despite a decrease in strength, are still strong enough for fulfilling their purpose—the limit of 35 N ± 5 N from which the tissues are damaged [38] being far exceeded. However, this decrease in the mechanical properties should be taken into account when designing 3D-printed medical instruments.

Not only acetone treatment was reported as decreasing the mechanical properties. Miguel et al.’s research focused on Nylon specimens with two raster angles (0°/90°, ±45°), 0.1 mm layer thickness and two coatings: polyurethane elastomer and liquid silicone [31]. Tensile and compressive properties were measured. Both types of coatings were found to decrease the mechanical properties. This conclusion can be added to the one discussed in Section 3.1 regarding Leite et al.’s study on surface post-processing treatments effect on compressive and tensile properties of 3D-printed ABS samples [29]. 

**Figure 11:**
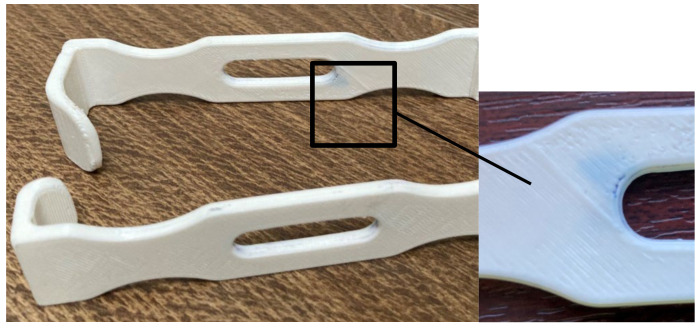
Retractors treated with acetone and decontaminated.

As mentioned in Section 2, the retractors were manufactured using the settings that provided least sealing, but at a shorter manufacturing time and cost (0.4 mm layer thickness vs. 0.1 mm layer thickness; 1 perimeter vs. 2 or 3 perimeters; and lines infill vs. gyroid infill). Three retractors were treated with acetone and then decontaminated (disinfected, washed, and sterilized), and three of them were used as controls. The presence of the slot in the retractor design produced air gaps at the intersection of the rasters with the slot perimeter. These were not filled in by the material melted by the acetone vapors during the exposure time. A longer action of the acetone vapors could have closed this defect eventually, but the retractors shape and dimensional stability would have been affected. Moreover, the slot of the retractor was oriented along the acetone vaporization direction and its interior was not smoothed out well. After the disinfection and washing, the acetone treated retractors were weighed and the mass gain was not significant. This shows that the post-processing was efficient in stopping the disinfectants infiltration within the retractor, but the blue stains on the surface (Figure 11) show that there are zones were the vapors surface treatment was not sufficient. It took 30 min for disinfectant to spread in the retractor as shown. Washing and brushing the retractors surfaces removed some of the stains, but not the one evidenced in Figure 11.

A solution to this problem can be to wipe the zones with defects (for instance, the perimeter to raster voids on the top/bottom surface of the retractor) with a cloth impregnated with acetone until they are completely smoothed. It is important to note that the more complex the design of a medical device, the more prone is to 3D printing defects. Additionally, it is more difficult to decontaminate it and smooth its surface, specific maneuvers being required as mentioned in Section 2.2. This is another aspect to be considered by the designer.

Acquiring knowledge on the behavior of the 3D-printed medical instruments or devices subjected to typical usage conditions (medical decontamination meaning disinfectant immersion, cleaning, and sterilization; contact with the disinfectants and body fluids; combined mechanical loads; repeated sterilization, etc.) is mandatory for safety reasons. This information is valuable for engineers who have to consider these specific aspects in the design (dimensions, small features, and geometrical complexity) and in 3D printing (by selecting optimal process parameters, material and post-processing method).

Starting from a characteristic of the 3D prints, namely the open porosity caused by the formation of external pores between the layers and internal voids determined by the threads’ deposition paths, a main concern is related to the 3D-printed ABS medical instruments permeability to fluids, which can pose safety risks to the patients. In this context, this research was focused on investigating how the 3D printing process parameters are influencing the infiltration rate of disinfectants, if the disinfectants are degrading 3D prints mechanical properties, and if the cold vapors acetone treatment (a cheap and easy applicable post-processing method) can ensure the necessary sealing.

The results of the research provided answers to the three research questions mentioned in the introductory section as the main objectives of investigations:

- Research question 1: Is there a set of 3D printing parameters that can improve the sealing and reduce the fluid infiltration?

Experimental context: Three printing parameters were considered: layer thickness, number of perimeters, infill pattern. Samples’ mass gain and absorption rate caused by disinfectant infiltration were determined for different parameters settings.

Answer: It was found that the layer thickness is the most influential parameter on the permeability (the smaller the thickness, the better the sealing), while the infill pattern is the least important one.

- Research question 2: Are the mechanical performance of 3D-printed specimens degraded after immersion in disinfectants and sterilization?

Experimental context: The compressive and tensile properties of saturated samples were determined.

Answer: The experimental results showed that the disinfectant is not degrading the material and it is not significantly influencing the tensile behavior. During the compression testing with load applied along the build direction, the saturated samples with lines infill are stiffer than the samples with gyroid infill.

- Research question 3: Is the acetone treatment a solution for sealing the 3D-printed ABS reusable medical instruments and not degrading the mechanical performances in corroboration with the medical decontamination?

Answer: Cold vapors acetone treatment reduces the open porosity. This post-processing method applied to 3D-printed retractors followed by medical decontamination was found to degrade the mechanical properties. However, this reduction is not a dramatic one, as the surface treatment with cold acetone vapors is not degrading material bulk properties if the exposure is not extensive. At the same time, the more complex the geometry of the medical item, more probable is the occurrence of printing defects, and the harder it is to provide proper cleaning of all zones and to eliminate the pores from its external surfaces by post-processing. This is an aspect to be taken into account by the designer, along with the mechanical properties decrease associated with the surface treatments.

## 4. Conclusions

The main conclusion of this research is that the 3D prints used in contact with the patient should be post-treated for eliminating the open porosity and ensuring a safe use. Cold vapor acetone treatment can be efficient if applied so as not to degrade the print and its dimensional stability, and to ensure that vapors smooth all the surfaces. The experimental results suggest the need of both reducing the porosity by parameter settings and applying post-treating on the medical instrument surfaces.

The results of the absorption tests proved that no manufacturing settings can provide enough sealing against fluid intake. However, some parameter settings can improve the sealing, in this sense the layer thickness being the most important factor. As the printing time and cost are influenced by the infill density, in many practical situations the prints are manufactured with 50–70% densities, meaning that more air gaps will be formed inside the part and more infiltration will occur. As such, the sealing of 3D prints will be more important.

The experimental outcomes also showed a decrease in the mechanical performance of 3D-printed ABS instruments treated by acetone cold vapors and then medically decontaminated in comparison to the control prints.

Further research will be focused on 3D prints sterility analysis as a function of infill density when the prints are not sealed by the surface treatments.

## Figures and Tables

**Table 1:** Printing parameters and their levels for compression specimens.

Fixed Parameters	Variable Parameters		
Material: ABS Extrusion temperature: 230 °C Bed temperature: 70 °C Infill density: 70% No adhesion Infill overlap: 30% Flow rate: 100% Top/bottom: 2 layers Line width: 0.4 mm Printing speed: 50 mm/s	Perimeters		
	1	2	3
	Layer thickness		
	0.1 mm	0.2 mm	0.4 mm
	Infill pattern		
	Lines at ±45°		Gyroid

**Table 2:** 3D-printed specimens for compression testing.

Cubic Specimens	Codification	No. of Perimeters	Layer Thickness (mm)	Infill Pattern
S1	S1_01_L	1	0.1	Lines
S2	S1_01_G	1	0.1	Gyroid
S3	S1_02_L	1	0.2	Lines
S4	S1_02_G	1	0.2	Gyroid
S5	S1_04_L	1	0.4	Lines
S6	S1_04_G	1	0.4	Gyroid
S7	S2_01_L	2	0.1	Lines
S8	S2_01_G	2	0.1	Gyroid
S9	S2_02_L	2	0.2	Lines
S10	S2_02_G	2	0.2	Gyroid
S11	S2_04_L	2	0.4	Lines
S12	S2_04_G	2	0.4	Gyroid
S13	S3_01_L	3	0.1	Lines
S14	S3_01_G	3	0.1	Gyroid
S15	S3_02_L	3	0.2	Lines
S16	S3_02_G	3	0.2	Gyroid
S17	S3_04_L	3	0.4	Lines
S18	S3_04_G	3	0.4	Gyroid

**Table 3:** 3D-printed parameters for tensile specimens.

Tensile Specimens Codification	Process Parameters
T_1_01_L_best	Layer thickness = 0.1 mm Infill pattern = lines Number of perimeters = 1
T_2_04_G_least	Layer thickness = 0.4 mm Infill pattern = gyroid Number of perimeters = 2

**Table 4:** Compression results of the disinfected ABS specimens.

Specimen	Mass at Saturation (g)	Modulus of Elasticity (MPa)	Mass Normalized Modulus of Elasticity	Compressive Yield Strength (MPa)
S1_01_L	1.329	3771.49	2837.84	28.38
S1_01_G	1.318	3463.72	2628.01	24.21
S1_02_L	1.337	3631.71	2716.31	25.73
S1_02_G	1.355	3100.39	2288.11	22.44
S1_04_L	1.334	2135.39	1600.74	18.78
S1_04_G	1.395	2099.72	1505.18	18.15
S2_01_L	1.349	5016.32	3718.55	33.67
S2_01_G	1.407	4918.42	3495.68	30.81
S2_02_L	1.277	4080.17	3195.12	26.61
S2_02_G	1.393	3229.39	2318.30	20.99
S2_04_L	1.381	2291.97	1659.65	16.77
S2_04_G	1.377	2041.26	1482.40	16.35
S3_01_L	1.422	4054.70	2851.41	26.89
S3_01_G	1.407	3072.50	2183.72	23.49
S3_02_L	1.452	3494.14	2406.43	25.51
S3_02_G	1.436	2941.79	2048.60	23.80
S3_04_L	1.428	2246.76	1573.36	21.95
S3_04_G	1.420	1958.60	1379.30	15.02

## Data Availability

The data that support the findings of this study are available from the corresponding author upon reasonable request.
